# Polyostotic vertebral osteomyelitis and myositis in a dog with *Anaerobiospirillum succiniciproducens* bacteraemia

**DOI:** 10.1111/jsap.13814

**Published:** 2024-12-09

**Authors:** T. Liatis, A. Skarbek, C. Jones, S. Wyatt

**Affiliations:** ^1^ Queen Mother Hospital for Animals Royal Veterinary College Hatfield UK

**FIG 1:**
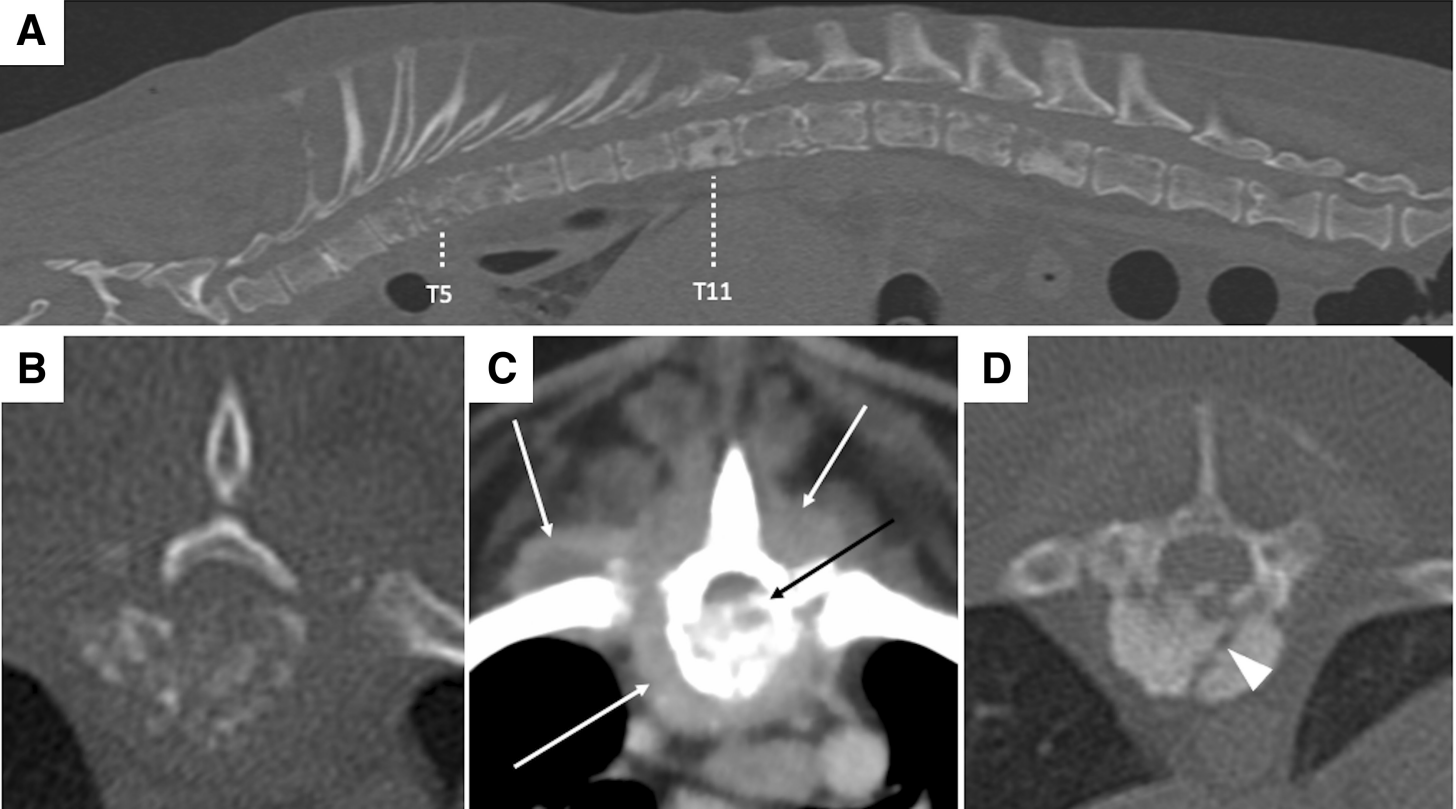
CT of the vertebral column in a dog diagnosed with polyostotic vertebral osteomyelitis and myositis. (A) Sagittal plane bone window reconstruction of the thoracolumbar vertebral column, window width 4500, window level 450. (B,D) Transverse plane bone window reconstructions at the level of T5 and T11, respectively, window width 2897, window level 543. (C) Transverse plane soft tissue reconstruction at the level of T5, window width 400, window level 40. (A) There are regions of bone lysis and sclerosis of multiple vertebral bodies. The T6 vertebral body has loss of normal architecture and indistinct margins with the adjacent vertebral bodies, it is markedly shortened suggestive of a compression fracture. (B,C) The T5 vertebral body is markedly lytic. There is loss of normal vertebral body architecture with mineral and soft tissue attenuating, contrast‐enhancing material within the vertebral canal causing spinal cord compression (image C – black arrow). The paravertebral soft tissues, including the multifidus, rotatores, iliocostalis and longus colli muscles (image C – white arrows), as well as the longissimus thoracis (not included in this image) are thickened and heterogeneously attenuating and contrast enhancing. (D) The T11 vertebral body is markedly sclerotic with a non‐displaced, complete fracture (white arrow head).

A 12‐year‐old male neutered Dachshund presented following a 3‐week history of lethargy, hyporexia, pyrexia and spinal pain, which acutely progressed to non‐ambulatory paraparesis. Amoxicillin‐clavulanate 20 mg/kg intravenously was administered once 1 day prior. The dog was diagnosed with sterile panniculitis 4‐years prior and has since been maintained on oral prednisolone (currently 0.8 mg/kg once on alternate days). Physical examination revealed pyrexia and generalised poor muscling. Neuroanatomical localisation was consistent with a T3‐L3 myelopathy. Haematology revealed inflammatory leukogram, and anaemia (28.5%, reference intervals [RI]: 37% to 55%), whilst serum biochemistry revealed increased C‐reactive protein (167.6 mg/L, RI <10 mg/L). CT revealed polyostotic osteolytic lesions affecting multiple vertebrae (Fig 1), the right eleventh rib, iliac wings and left scapula. Additionally, there were thickened heterogeneously hyperattenuating and moderately enhancing paraspinal soft tissues and a cranial mediastinal lymphadenomegaly. Differential diagnoses included vertebral osteomyelitis (bacterial/fungal) or neoplasia (multiple myeloma/multifocal osteosarcoma). Urinalysis was unremarkable including negative Bence‐Jones proteins. CT‐guided cytology of T5 and L3 vertebrae and paraspinal muscles suggested neutrophilic inflammation, whilst hepatic and splenic cytology were unremarkable. Bone marrow cytology and biopsy from the right ilium revealed myeloid hyperplasia without evidence of microorganisms or neoplastic cells. Bacterial and fungal cultures from urine and bone marrow were negative. Blood culture was positive to *Anaerobiospirillum succiniciproducens* and a diagnosis of suspected bacterial vertebral osteomyelitis and myositis was made. Concurrent meningomyelitis cannot be excluded as cerebrospinal fluid analysis was not performed. Treatment with amoxicillin‐clavulanate for 12 weeks and multimodal analgesia was initiated, and prednisolone was discontinued. At 8‐weeks, the dog was comfortable but remained non‐ambulatory, and subsequently had a relapse of clinical signs (pyrexia, anorexia and marked spinal hyperaesthesia) 4 weeks after discontinuation of antibiotics. *A. succiniciproducens* is a rare anaerobic bacterium considered part of the normal gastrointestinal microbiota in dogs and it has been implicated in sporadic cases of bacteraemia, usually in immunocompromised human patients. In this case, chronic immunosuppression was proposed as a cause for opportunistic bacterial infection with a commensal species. Urine and bone marrow cultures may be negative due to prior use of antibiotics or their low sensitivity in regions with mild lesions and low microbial burden (e.g., iliac bone).

